# Nanomodulation of Macrophages in Multiple Sclerosis

**DOI:** 10.3390/cells8060543

**Published:** 2019-06-05

**Authors:** Frances K. Nally, Chiara De Santi, Claire E. McCoy

**Affiliations:** Molecular and Cellular Therapeutics, Royal College of Surgeons in Ireland, 123 St Stephen’s Green, 2 D02 YN77 Dublin, Ireland; francesnally@rcsi.ie (F.K.N.); chiaradesanti@rcsi.ie (C.D.S.)

**Keywords:** multiple sclerosis, experimental autoimmune encephalomyelitis, macrophage polarisation, monocytes, microglia, inflammation, nanoparticle, microparticle, drug delivery, CNS

## Abstract

Multiple Sclerosis (MS) is a chronic demyelinating autoimmune disease primarily affecting young adults. Despite an unclear causal factor, symptoms and pathology arise from the infiltration of peripheral immune cells across the blood brain barrier. Accounting for the largest fraction of this infiltrate, macrophages are functionally heterogeneous innate immune cells capable of adopting either a pro or an anti-inflammatory phenotype, a phenomenon dependent upon cytokine milieu in the CNS. This functional plasticity is of key relevance in MS, where the pro-inflammatory state dominates the early stage, instructing demyelination and axonal loss while the later anti-inflammatory state holds a key role in promoting tissue repair and regeneration in later remission. This review highlights a potential therapeutic benefit of modulating macrophage polarisation to harness the anti-inflammatory and reparative state in MS. Here, we outline the role of macrophages in MS and look at the role of current FDA approved therapeutics in macrophage polarisation. Moreover, we explore the potential of particulate carriers as a novel strategy to manipulate polarisation states in macrophages, whilst examining how optimising macrophage uptake via nanoparticle size and functionalisation could offer a novel therapeutic approach for MS.

## 1. Introduction

Multiple Sclerosis (MS) is a chronic inflammatory disease of the Central Nervous System (CNS), affecting an estimated 2.3 million people worldwide [1]. On average, disease onset and diagnosis occurs between the ages of 20 and 40, with occurrence 2–3 times higher in females than males, making MS the most common neurologically disabling disease in young adults [2]. Pathologically, the disease is characterised by the appearance of focal lesions in white and grey matter of the brain and spinal cord, indicative of extensive loss of oligodendrocytes and myelin sheath. Owing to the distribution of such lesions, clinical presentation is variable among patients and can include impairments in motor, sensory and cognitive functions, as well as pain and fatigue [3]. MS is a complex disease with incompletely understood aetiology with contribution from genetic predisposition [4], as well as environmental risk factors, including geographical location, Epstein Barr virus infection, human cytomegalovirus, lack of vitamin D and circadian rhythm disruption [5,6]. In 80–85% of newly diagnosed individuals, symptoms occur on a relapsing remitting basis (RRMS), with roughly two thirds of these go on to develop secondary progressive disease (SPMS) [7]. Primary progressive disease (PPMS) occurs in a smaller proportion of individuals [8]. 

Despite the fact the instigating factor in disease pathogenesis of MS remains elusive, plaque formation and disease symptoms are widely accepted as the result of immune cell infiltration, with the release of cytokines and inflammatory mediators leading to inflammation, myelin destruction, oligodendrocyte loss and eventual axonal degeneration [9]. While there is a complex immune pathology at play with contributions from many immune cell types, MS has been traditionally viewed as a T-cell mediated disease [10,11,12]. While initial discussion centred on CD4+ T-cells subsets, the essential role of CD8+ T cells has been more recently discussed [11,12,13], as well as that of B lymphocytes [14,15]. However, emerging and accumulating evidence has highlighted a role for infiltrating monocytes and macrophages in human MS pathology, as comprehensively discussed in [16] and as further discussed in this review. These cells are the most predominant cell type in patient lesions [17,18,19,20], with their presence correlating with both demyelination [21,22] and axonal damage and degeneration [20,22,23,24]. 

Experimental autoimmune encephalomyelitis (EAE) is among the most frequently used models to study the demyelinating and immune pathology of MS [25]. EAE can be induced by direct immunization of myelin antigens PLP, MOG or MBP in Complete Freunds Adjuvant, or by the transfer of isolated activated CD4+ T cells [26], or less commonly by CD8+T cells to a naïve animal [27,28], which avoids the immunological consequences of adjuvant administration [29]. While disease course is variable, dependent upon both animal species and strain, and on the inoculating myelin antigen [26], overall pathology in this model is largely driven by CD4+ T helper (Th)1 and Th17 T cell subsets [25,30]. This, coupled with the fact that demyelination and lesion formation occur predominantly in the spinal cord rather than the CNS, indicates that EAE does not fully recapitulate human MS pathology [25,29,31]. Nonetheless, the utility of the EAE model is exemplified in the discovery and development of some of the current approved therapies, namely Glatiramer Acetate [32], Natalizumab [33], Fingolimod [34] and has been used to further elucidate the mechanism of action of others, including Alemtuzumab and Dimethyl Fumarate [35]. Moreover, the use of toxin induced models such as lysolecithin, cuprizone and ethidium bromide (EtBr) are seeing increased popularity, enabling different aspects of MS disease pathology to be addressed [35]. While there is no model available that can recapitulate the entirety of the molecular and cellular events involved in MS, the use of animal models has been invaluable to our current knowledge of mechanisms at play in MS, including understanding the role of macrophages in disease pathology, and are essential for use in preclinical models [36].

In this review, we examine this role played by macrophages in MS and MS animal models, and explore the potential for the use of nanotechnology in developing macrophage-centred therapeutics for preclinical efficacy in MS.

## 2. Macrophages in MS

### 2.1. Resident vs Infiltrating Macrophages

Macrophages are a highly plastic, highly diverse population of cells, with a multifaceted role in the normal immune response as well as in disease. Macrophages are professional phagocytes, and are the most numerous cells found in CNS lesions in both human MS and EAE models [17,21,37]. In the context of disease, these macrophages are a mixed population in terms of lineage, capable of arising from both CNS resident glial cells and infiltrating monocytes. Belonging to the former, microglia are the resident macrophages in the CNS parenchyma, with an essential role in neurological function and immunosurveillance under homeostatic conditions [38]. CNS resident macrophages are notable among myeloid populations; they arise from a distinct embryonic yolk sac population that enter the CNS prior to blood-brain barrier (BBB) closure and do not undergo replacement by hematopoietic precursors throughout life [39,40,41]. Non-parenchymal CNS resident macrophages, which include perivascular, meningeal and choroid plexus macrophages, also arise from embryonic populations and, with the exception of those in the choroid plexus, undergo little replacement by blood borne monocytes [42]. Monocytes and macrophages of peripheral origin are normally not present in the parenchyma of the healthy CNS, but are recruited in response to EAE induction and can be found in lesions in both EAE and MS pathology [17,21,37].

There is substantial evidence that the infiltrating monocytes, rather than resident microglia, play a more prominent role in driving disease pathogenesis. For example, microglial activation appears to be a key early feature, distinct from monocyte entry, and does not ensure disease onset. In a model where reduced sensitivity to pharmacogenetic depletion resulted in microglial paralysis, EAE onset was delayed, with reduced clinical scoring, diminished monocyte infiltration and reduced myelin and axon destruction [43]. In other studies, microglial activation can be identified both prior to peripheral infiltration and appearance of symptoms, as well as in animals that fail to progress beyond the initial stage of EAE [44,45]. This suggests that although microglia play an important role in the initiation of disease in EAE, the continued progression of the disease is largely due to their role in monocyte recruitment. Monocyte depletion prior to symptom development delayed EAE onset and resulted in less severe clinical scoring [46,47,48] while depletion post-onset showed inhibited disease progression [46,49]. Furthermore, it has been demonstrated that in early disease, rising numbers of peripheral monocytes correlate with the severity of clinical scoring [45,50], with this infiltration correlating with progression to paralysis [45]. Moreover, observations from histological studies of human samples point to a role for microglia at similar early stages of lesion formation, noting their activation in normal appearing white matter prior to peripheral infiltration and myelin destruction [51,52,53,54]. The remainder of this review will focus on infiltrating monocytes and how they contribute to disease progression as well as to its resolution. We illustrate how this dual capacity of infiltrating monocytes could be manipulated as a novel therapeutic approach for MS.

### 2.2. A Dual Role for Macrophages in MS

Monocytes are mobilised by chemokine signalling and traverse the blood brain barrier in response to the induction of EAE, with the chemokine receptor CCR2 and ligand CCL2 particularly well explored in this context. A number of studies have demonstrated that the absence of CCR2, or one of its major ligands, CCL2, results in diminished or absent EAE development [55,56,57,58]. Crucially, this effect has been shown to be most prominently mediated through CCR2 engagement in the myeloid compartment [59]. CCR2 expression in monocytes occurs in a population primarily defined by high expression of Ly6c in mice that traffic to sites of inflammation [60]. Antibody mediated targeting of the CCR2+Ly6C^hi^ monocytes in the periphery during peak disease resulted in markedly reduced clinical scoring, while had no observable effect during the ‘priming’ phase of disease between EAE induction and onset [59]. In a relapsing model of EAE, this CCR2+Ly6C^hi^ population increase markedly in the blood prior to relapse in a GM-CSF dependent manner [61]. GM-CSF is a cytokine produced by Th subsets, and is essential to disease development [62,63]. Interestingly, a conditional receptor deletion strategy by Croxford and colleagues has shown the CCR2+ monocyte population to be the crucial facet of GM-CSF mediated pathogenesis in EAE [64]. 

While monocyte populations are somewhat differently defined in humans, consisting of CD14++CD16− classical monocytes, CD14+CD16++ non classical monocytes and CD14++CD16+ intermediate subsets (outlined in [65]), changes to the monocyte populations in MS patients has been described. Increases in the non-classical populations in circulation have been shown in both RRMS and PPMS [66,67,68]. While this CD14+CD16++ population is normally low in CCR2 expression [69], its expression has been shown to be significantly upregulated in this population of monocytes from the PBMC’s of MS patients [68]. CCL2 expression has also been noted in both MS plaques and in the CSF of patients with ongoing disease [70,71,72,73]. There is ongoing debate as to whether this CCR2 mediated chemotaxis is as important in MS as it is in animal disease models [74,75,76], nonetheless, the potential for a therapeutic CCR2 targeting antibody is under investigation [77].

**Figure 1:**
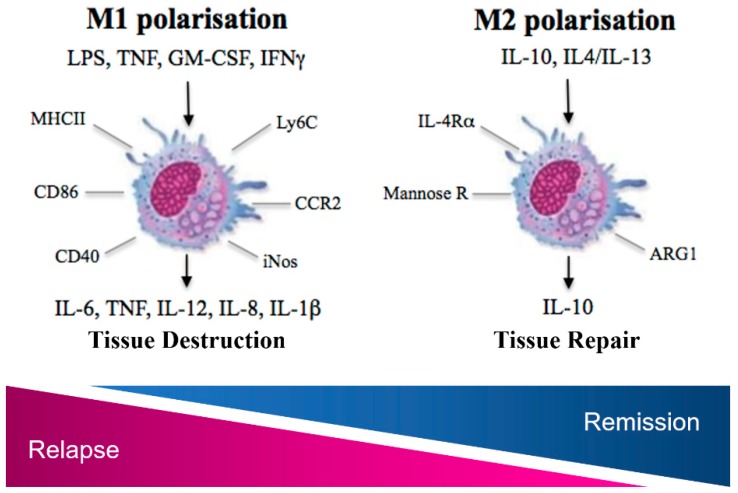
Schematic illustrating canonical M1 and M2 polarised macrophages that result in tissue destruction and tissue repair in the CNS in MS and experimental autoimmune encephalomyelitis (EAE). Agonists, cell surface markers, receptors and typical cytokines released are also highlighted.

Following tissue entry into the spinal cord or CNS parenchyma, monocytes become activated, differentiate, becoming myeloid dendritic cells (DCs) or macrophages. With regard to macrophages, because this activation occurs in response to a variety of signals, there is considerable functional heterogeneity [78]. In EAE, T cell produced GM-CSF, IFNγ and TNFα cause polarisation to a pro-inflammatory phenotype or M1-like phenotype, with cells arising from CCR2+Ly6C^hi^ monocytes expressing high levels of MHCII, IL-6, IL-12p40, iNOS and inflammasome products IL-1α and IL-1β [59,61,64] (Figure 1). These macrophages facilitate damage to the CNS in a number of ways, for example, MHCII, CD86 and CD40, as well as IL-12 are essential for antigen presentation and persistent T-cell activation in EAE [79,80]. The release of other pro-inflammatory cytokines, including TNFα, IFNγ and IL-6, as well as proteases, reactive oxygen species (ROS), reactive nitrogen species (RNS) establishes an inflammatory microenvironment that facilitates damage to the myelin sheath and the surrounding cells [81]. As well as their general role in the inflammatory cascade, there is strong evidence that macrophages directly mediate axon damage, an aspect of MS pathology that is attributed to permanent, progressive symptoms [82]. ROS and RNS of macrophage/microglial origin have been implicitly shown to cause axonal degeneration in EAE [83], with monocyte-derived macrophages in particular associated with direct axon contact [84]. Similarly, in human disease, axon loss in lesions is most prominently correlated with pro-inflammatory activity in macrophages [19,22,23,24], evident in both relapsing, primary progressive and secondary progressive disease [20]. Higher iNOS expression and activity is evidenced in both circulating patient monocytes as well as MS brain tissue versus healthy controls [85,86], and is significantly correlated with axon densities in lesions [19]. Crucially, iNOS expression in the CNS co-localises with CD64+ macrophages and is associated with the presence of nitrotyrosine and MBP fragments, highlighting the contribution of NO mediated damage at these sites [22].

While the pro-inflammatory population predominates in early disease, a gradual shift occurs through intermediate activation states [54,87,88]. By the remission phase of EAE, cells have a more alternatively activated or M2-like phenotype as clearly evidenced by an elegant fate tracing study labelling cells expressing iNOS and Arginase-1, canonical markers of M1-like and M2-like function, respectively [87]. Importantly, this functional shift can also be observed in MS patient lesions, with myelin-laden macrophages expressing high levels of M2 associated CD163 and CD206 [54], with a recent study highlighting the presence of CD206+ cells in inactive lesion centres, while iNOS expression was associated with areas of active pathology at lesion edges [88].

M2 polarisation can be driven by IL-4/IL-13 and IL-10 in vitro, with the latter driving a distinct transcriptional signature and overall, a more profound inhibition of pro-inflammatory processes [78,89]. IL-10 upregulation in the CNS in the recovery phase of the EAE model has been demonstrated [90,91], while the administration of IL-10 expressing mesenchymal stem cells is suppressive to disease development, with a capacity to suppress bone-marrow derived dendritic cell (BMDC) antigen presentation in vitro [92]. Macrophage IL-10 production is a general characteristic of alternative activation, with autocrine signalling resulting in downregulation of MHCII and co-stimulatory molecules, suppressing antigen presentation and hampering CD4 T cell responses [89,93,94]. Suppression of the iNOS mediated respiratory burst also occurs, with the IL-4 driven upregulation of Arginase-1 [89], which depletes NO precursor arginine and drives the polyamine pathway, associated with collagen synthesis and tissue repair in the damaged CNS in animal models [95]. IL-4 is also associated with upregulation of scavenger receptors and enhanced endocytotic ability [89], facilitating clearance of myelin debris as evidenced in human MS lesions [96]. Moreover, IL-10 and IL-4 immunoreactivity has been shown in human MS brain tissue in active demyelinating lesions and at the rim in chronic active lesions, with receptors for these cytokines highly expressed by macrophages in parenchymal and perivascular areas [97].

This tissue reparative role, coupled with their inflammation limiting capacity, positions M2-like macrophages as key effectors in disease remission. Supporting this, the adoptive transfer of M2 polarised cells is beneficial in preventing EAE development [98,99]. There is evidence of a pro-repair action that can be mediated without CNS entry of adoptively transferred cells, suggesting that this effect may be mediated in the peripheral environment [100]. A more direct effect of the M2 phenotype on remyelination within CNS lesions has also been indicated. In toxin-induced demyelination, decreased oligodendrocyte precursors were linked to the loss of macrophage secreted growth factors as a result of depletion [101]. Additionally, Miron and colleagues have demonstrated a role for M2 macrophages in murine oligodendrocyte differentiation that is diminished by M2-specific depletion within lesions and highly dependent on M2-associated TGF-β family signalling molecule, Activin-A [102]. Interestingly, increased TGF-β is observed in blood cell cultures and in CSF of patients in remission compared to those with active disease [103,104]. The studies in animal models implicating macrophages in repair are additionally complemented by observations in human MS patients, where in resolving lesions with remyelination, macrophages persist, coexisting with this repair process and displaying different morphology and staining patterns than those observed in acute disease [105].

As outlined, we clearly see a dual role for macrophages in MS models and human disease owing to their polarisation potential, at the outset contributing substantially to disease pathogenesis, while studies in animal models highlight an essential reparative role, with evidence to support similar mechanisms in human MS. Some of the aforementioned studies illustrate that by depleting or increasing these populations experimentally, the outcome of EAE can be substantially altered. Thus, modulating these populations to minimise pro-inflammatory or M1-like and favour M2-like polarisation may hold potential for therapeutic translation in MS.

### 2.3. Current Therapeautics and Their Impact on Monocytes and Macrophages

While MS remains incurable, there has been considerable development over the last 25 years in terms of available disease modifying therapies (DMTs). First introduced in the mid-1990s, there are over 10 FDA approved treatments presently available (summarised in Table 1) which show efficacy in reducing disease relapses in RRMS patients [106]. Despite the advancement, Interferon Beta (IFNβ) and Glatiramer Acetate (GA), the first two drugs to be introduced, remain the “first line” therapies for MS owing to their relative safety and proven efficacy [106,107]. Both drugs are administered by self-injection and function in an immunomodulatory capacity. Oral DMTs available for RRMS include immunosuppressives Fingolimod and Teriflunomide (TFM), as well as immunomodulatory Dimethyl fumarate (DMF). In addition, a number of monoclonal antibody-based therapeutics for RRMS have emerged, including natalizumab and alemtuzumab. While highly efficacious in preventing relapse and disability, these newer treatments carry high risk side effects including progressive multifocal leukoencephalopathy (PML) and development of secondary autoimmune diseases, respectively [108,109]. Moreover, Daclizumab, a monoclonal antibody against the alpha subunit of the IL-2 receptor has been recently removed from the market due to reports of encephalitis development [110].

Broadly, these therapies act by either altering T-cell responses (IFNβ, GA, DMF), inhibiting lymphocyte trafficking (Fingolimod, Natalizumab) or depleting lymphocyte populations (Alemtuzumab, Ocrelizumab, TFM, Mitoxantrone). How these therapies impact on monocyte and macrophages, however, has been less explored. Below and in Table 1 we consider evidence of any direct action of DMTs on monocyte and macrophage populations, which may contribute to their respective therapeutic efficacies.

#### 2.3.1. Interferon-β

IFNβ, a type 1 interferon, is an anti-inflammatory cytokine and was the first available DMT for the treatment of MS. In addition to affecting T and B lymphocyte function and reducing BBB transmigration [111,112], IFNβ exerts effects cells of the innate immune system in the context of MS. Of note, two studies demonstrate a key role of IL-27 production by DCs and macrophages in suppressing Th17 T cell mediated responses in EAE models [113,114]. An effect of IFNβ treatment on human monocytes has also been documented, with monocytes from treated patients shifting towards a CD14++CD16+ intermediate phenotype [66]. Notably, patient monocytes produce less IL-1β in response to inflammatory stimuli [143], and show significantly reduced production of IL-8 and CCL2 after ex vivo T cell activation [144]. In terms of IFNβ on macrophage and monocyte polarisation, a study by Liu and colleagues show enhanced sensitivity to IL-10, a driver of the M2 phenotype, through upregulation of the IL-10 receptor in both human monocytes and macrophages [115]. In conjunction with the increased serum IL-10 levels seen in IFNβ treated MS patients [145,146], this indicates IL-10 modulation of macrophages and their monocyte precursors may occur in response to IFNβ treatment.

#### 2.3.2. Glatiramer Acetate

GA is a synthetic copolymer of lengths 50 to 90 residues of randomly arranged L-tyrosine (Y), L-glutamic acid (E), L-lysine (K), L-alanine (A), with its efficacy chiefly credited to its ability to modulate peripheral T cells towards a Th2 phenotype and increase the Treg population [117,118]. The effects of GA on the myeloid cell population are also believed to contribute to its therapeutic efficacy. This effect was initially demonstrated on human and animal cells in vitro, with GA treated monocytes showing decreased TNFα and cathepsin B levels in response to inflammatory stimuli, as well as increased production of anti-inflammatory IL-10 [147,148]. Similar findings were recapitulated in a number of studies utilising isolated monocytes from GA treated patients, showing decreased TNFα, IL-12 and IL-1β in conjunction with increased IL-10, TGF-β and IL-1 receptor antagonist [119,120,121]. This cytokine shift in GA-treated monocytes is primarily explored in terms of the effects of antigen presentation by myeloid lineage cells on the T-cell response. The effects seen are consistent with type 2 antigen presenting cells, which induce development of Th2 responses. Interestingly, GA has been shown to increase phagocytosis in both rat microglia and MS patient monocytes [122,123] with debris clearance necessary for remyelination [149]. Monocyte modulation may be among the most long-lived responses to GA treatment, with a study showing increased anti-inflammatory monocytes as one of two significant changes in the leukocyte population that prevail following treatment periods of up to 16 years in MS patients [150].

#### 2.3.3. Dimethyl Fumarate

DMF is an immunomodulatory drug originally used for psoriasis treatment, but is also therapeutically useful in the treatment of RRMS, resulting in diminished Th1 responses [151]. It is a known activator of the anti-inflammatory transcription factor Nrf2, as well as interfering in TLR signalling pathways upstream of NFkB activation [152,153,154,155]. The immunomodulatory function of DMF extends to cells of the myeloid lineage, in EAE resulting in reduced monocyte infiltration and microglia with a more alternatively or ‘M2′ activated phenotype [132,133]. In murine monocytes, DMF treatment increases myeloid derived suppressor cells and results in an alternative activation profile in monocytes consistent with Type II APCs, while in macrophages decreases iNOS and TNFα expression, with concomitant increases in Arg1 and IL-10 [135,136]. Similar effects on monocyte populations have been demonstrated in DMF treated MS patients, with peripheral monocytes showing reduced levels of mir-155, a micro-RNA associated with major pro-inflammatory effect [125], while in vitro treated human monocytes showed suppressed TNFα, IL-6 and IL-10 responses to a pro-inflammatory stimulus [125]. Furthermore, it has been shown the DMF treatment results in reduced expression of MHCII molecules and NFkB in human myeloid DCs and a concomitant reduction in their capacity to activate T cells [156]. Interestingly, it has recently been shown that DMF has a profound effect on cell metabolism, blocking the glycolysis pathway by inhibition of glyceraldehyde 3-phosphate dehydrogenase (GAPDH) in murine macrophages [134]. This may contribute to the mechanism by which DMF promotes M2-like macrophages, as a preference for glycolysis is associated with M1 macrophages, while M2 macrophages see higher levels of oxidative phosphorylation [134,157].

#### 2.3.4. Fingolimod

Fingolimod is an antagonist of sphingosine 1 phosphate receptor, which functions to inhibit leukocyte passage out of the lymph nodes. In monocytes isolated from Fingolimod treated patients, alteration in cytokine production has been observed with a reduction in pro-inflammatory cytokines such as TNFα, IL-1β and IL-6 [127,128,129]. In addition, the capacity of fingolimod treatment to result in an M2 polarisation of microglia has been demonstrated in a murine model of stroke [126]. Similarly to MS patients treated with DMF and Natalizumab, Fingolimod also results in a reduction of pro-inflammatory mir-155 in circulating monocytes [125].

## 3. Nanoparticles and Microparticles in MS

The goal of most current MS therapies is dampening the immune response in the CNS, reducing the number and the severity of relapses and lesions in MS patients. Such treatment raises concerns due to the associated chronic, non-specific immunosuppression, which may pose a serious risk in the medium/long term [158]. Coupled with the fact that DMTs are not effective in all patients, this underlies an urgent need for the development of novel therapeutic strategies to overcome these issues. In recent years, nanotechnology has emerged as a promising method of drug delivery offering many advantages over conventional delivery mechanisms. Nanoparticle and microparticle carriers are colloidal particles, where sizes ranging from 10–1000 nm are generally designated nanoparticles (NPs), while those ranging from 1–250 μm are designated microparticles (MPs) [159]. NPs and MPs can be prepared from a wide range of materials; among the most popular are lipid and polymer-based nanoparticles including poly-lactide co glycolic acid (PLGA) and chitosan [160]. Such carrier systems offer the ability to deliver otherwise challenging molecules, such as nucleic acid and low bioavailability drugs and facilitate controlled release. In addition, nanotechnology can mediate cell specific delivery and passage across biological barriers like the BBB through targeting by carrier type, size, surface charge, and conjugation of specific targeting ligands. This minimizes interaction with non-target cell types and can reduce the amount of drug required for sufficient accumulation in target cells. As outlined above, macrophage manipulation may offer novel opportunities in the treatment and management of MS. Macrophages are attractive targets for NP/MP -mediated delivery, usually seeing high uptake due to their phagocytic nature. In the following sections, we will give an insight on the role that NPs/MPs could play in either improving the efficacy of existing drugs or helping the delivery of newly developed therapeutics in preclinical models of MS, with particular emphasis on targeting macrophages. Although the majority of studies were performed in animal studies, it holds much promise for their translation in MS; as it was previously mentioned that many of the approved MS drugs have been tested for safety and efficacy in EAE models, as is recommended by preclinical guidelines [36]. A number of NP/MP based studies in MS models with impact in macrophages are explored in Table 2.

### 3.1. Monocyte and Macrophage Depletion

The first studies to demonstrate NP/MP uptake by macrophages and efficiency in EAE were conducted as early as 1981, where intraperitoneal (IP) injections of silica quartz dust in rats was successful in lowering the clinical score in the EAE model by depletion of the peritoneal macrophage population [46]. Similar results were obtained by Huitinga and co-authors shortly after, where intravenous (IV) administration of mannosylated liposomes containing dichloromethylene diphosphonate (Cl2MDP) in rats selectively depleted circulating monocytes and macrophages in the spleen and the liver [47]. This was accompanied by a lower infiltration rate of monocytes in the CNS and subsequent improvement of the clinical score in EAE model. Further research provided more details about the mechanisms of action of the CI2MDP-liposomes, showing that the myelin sheath of treated mice was not affected, with the expression of iNOS and TNFα by macrophages dramatically inhibited [48]. Importantly, the authors showed that the liposomes impede the CNS infiltration specifically of monocytes but not T-cells, suggesting again a key role of these innate immune cells in the onset and progression of the disease in the EAE model [48].

In line with these early findings, Getts and colleagues have shown that empty carboxylated PLGA and other negatively-charged MPs injected IV were taken up specifically by monocytes and monocyte-derived macrophages via the macrophage receptor with collagenous structure (MARCO) [179]. These cells were then less able to migrate into the brain and accumulated for a short period in the spleen, where they underwent apoptosis, accompanied by a reduction in the clinical score of the EAE. However, the authors did raise concerns about using this drastic approach in disorders where monocyte-derived macrophages are important also for disease resolution, as is indicated in MS, underpinning the limitation of total depletion as a viable therapeutic strategy.

### 3.2. NP/MP and Antigen Specific Tolerance Induction

Tolerogenic nanoparticles (tNPs) are typically packaged with the antigen that elicits the abnormal immune response in autoimmune disorders. They exert their immunomodulatory function by selectively targeting professional antigen-presenting cells (APCs), such as macrophages and DCs, due to their intrinsic ability to internalise tNPs via endocytosis. APCs loaded with tNPs are then able to elicit an effective antigen-specific immune response, as elegantly reviewed in Kishimoto & Maldonado [180]. In the context of MS, tNPs are prepared by encapsulating myelin antigens into different carriers, where they are taken up by APCs which then efficiently trigger tolerance induction in autoreactive T cells in in vivo models. The typical cargo is the myelin antigen in one of its different forms, including myelin oligodendrocyte glycoprotein (MOG) [171,172,174,175,176], myelin basic protein (MBP) [173] and proteolipid protein (PLP) [168,178,181]. The carriers vary from study to study, and they include PLGA [168,171,174,178,181], acetalated dextran [175], poly(ε-caprolactone) [173] and gold [172], among others. Interestingly, coupling the myelin antigen with immunomodulatory agents like dexamethasone [175], IL-10 [174], 2-(1′Hindole-3′-carbonyl)-thiazole-4-carboxylic acid methyl ester (ITE) [172], or rapamycin [168,181] has shown efficacy in the EAE model.

Considering APCs are essential for mediating the tNP effect, it is not surprising that some studies have shown a reduction in EAE clinical score after tNPs administration, due to the specific contribution of macrophages. For example, IV injection of PLGA and polystyrene PLP-tNPs in EAE mice models were taken up specifically by macrophages via interaction with the scavenger receptor MARCO and delayed the onset or improved the progression of the disease [178]. Similarly, PLGA PLP-tNPs administered with rapamycin were shown to co-localise with macrophages in the spleen, although the precise effects of tNPs on macrophages were not fully elucidated [168]. Functionally, treatment of PLGA MOG-tNPs in EAE mice dramatically reduced the number of activated macrophages and microglia in the CNS and this contributed to the overall decreased disease severity, while also reducing the number of CD86+MHCII+ DCs in lymph nodes [176]. Collectively, these works show that macrophages play a fundamental role in the uptake of tNPs, improving clinical progression in the EAE model and illustrating a promising NP-mediated, macrophage directed therapeutic approach.

### 3.3. Cortiocsteroid Delivery

Although not considered DMTs, steroids (including methylprednisolone, dexamethasone and prednisolone) are often used as first line response to treat acute relapses in MS patients due to their potent and quick ability to close the damaged blood brain barrier and reduce inflammation in the CNS. However, they are characterised by side-effects due to high doses and systemic administration. This issue can be addressed by administering them as liposomal drugs, which lead to higher tissue concentrations in the inflamed target organ compared to an equivalent dose of the free drug. Several research groups showed evidence of this advantage by using different carriers to enhance the efficacy of steroids in the EAE model, with macrophages frequently shown to be the primary effectors of this response [162,163,164,165,182].

IV administration of Prednisolone (PL) and methylprednisolone (MPL) encapsulated in PEGylated liposomes has been investigated in EAE, resulting in improved clinical score when compared to free drug [163,164]. Interestingly, liposomes in these studies were shown to be taken up mainly by macrophages in the spinal cord of injured animals, and by microglia and astrocytes. The therapeutic efficacy of the PL and MPL liposomes was coupled with decreased blood brain barrier disruption, decreased macrophage and T-cell infiltration in the CNS, and reduced demyelination and axonal loss [163,164]. Similarly, an inorganic-organic hybrid NP loaded with glucocorticoid betamethasone (BMP-NP) was shown in vitro to be preferentially taken up by macrophages rather than T cells or B cells [162]. In vivo, macrophages from BMP-NPs treated mice polarised towards an anti-inflammatory phenotype to the same extent as free glucocorticoid drug dexamethasone (DEX), as shown by a reduction in MHCII and CD86 positive cells and in TNFα secretion [162]. While the number of infiltrating T cells did not change between empty-NP and BMP-NPs treated mice, the number of macrophages in the spinal cord were significantly reduced, suggesting again that these cells are the primary target of this therapy. As a proof of concept for human translation, the authors treated peripheral blood monocytes from healthy individuals with BMP-NPs and observed an increased expression of anti-inflammatory genes and lower levels of pro-inflammatory genes in qRT-PCR [162]. This work strongly supports the notion that polarisation of macrophages towards an anti-inflammatory state could be of therapeutic benefit in MS.

### 3.4. NPs/MPs and Current MS Disease Modifying Therapies

In order to improve their bioavailability and lower their adverse effect, recent studies have investigated encapsulation of orally administered DMTs into NP/MPs. For example, Kumar and collaborators have shown that the formulation of DMF-loaded nanolipidic carriers (NLCs) coated with vitamin-based neuroprotective molecules like tocopherol acetate cholecalciferol and retinol acetate improved the clinical score in a cuprizone-induced demyelination mouse model when given once daily orally [183]. Treatment with DMF-NLCs, especially when coated with the vitamin-based compounds, showed improved locomotor activity, motor coordination and balance compared to free DMF treated mice. Myelination status was also measured in brain slices, again with the DMF-loaded vitamin NLCs showing higher remyelination compared to free DMF [183]. Further histopathological analyses showed none of the stomach tissue damage in DMF-NLC treated mice that was observed in those treated with free DMF.

TFM, an orally administered DMT for MS, is often associated with hepatotoxicity, possibly due to its delivery route which leads to higher exposure of the drug within the systemic circulation [184]. TFM was loaded into NLCs subsequently combined with mucoadhesive and gelling agents in order to overcome mucociliary clearance and achieve efficient delivery via the nose to brain route [185]. TFM-NCL and mucoadhesive TFM-NCL (TFM-MNLC) were given orally and intranasally to rats in a cuprizone-induced demyelination model and recovery was assessed by an exteroceptive behavioural model. Although very preliminary, the results showed a trend of improved neurological function in rats treated intranasally versus orally without hepatic or renal biomarker elevation [185].

These studies illustrate the potential for the use of nanocarriers to improve efficacy and specificity of existing DMTs for MS. Given that DMTs can produce effects in monocytes and macrophages, coupled with the high NP/MP uptake generally seen in these cells, macrophages could play an unexplored role in the reduced disease severity in these models, which may be worth further investigation.

### 3.5. NPs/MPs and Novel Drugs

Besides the use of NPs/MPs as carriers for existing drugs and tolerogenic molecules, many research groups have exploited the typical features of NPs/MPs (low cell toxicity, longer bioavailability, ability to cross the blood brain barrier among others) to design and test novel therapeutic strategies in in vivo models. This has been evidenced in targeting a number of cell populations in MS. For example, the pro-remyelination factor leukaemia inhibitory factor (LIF) was encapsulated in PLGA NPs, and specifically targeted oligodendrocyte precursor cells (OPCs) by conjugating the NPs with NG2 chondroitin sulphate proteoglycan antibodies [186]. NG2-targeted LIF-NPs were able to induce significantly higher percentage of remyelinated fibres and to increase the myelin thickness per axon compared to non-targeted LIF-NPs, suggesting that the conjugation of LIF-NPs with NG2 antibodies, and thus the OPC-specific targeting, was critical for the observed therapeutic effect [186]. Similarly, another group investigated delivery to OPCs, this time intranasally administering short-interfering RNA (siRNA) in chitosan NPs in ethidium bromide induced demyelination [187]. The target, LINGO-1 is a transmembrane protein that suppresses myelination and axonal regeneration and the antibody Opicinumab/BIIB033 directed towards this mediator has been tested in clinical trials for MS with limited effectiveness, potentially due to low CNS penetration [188,189]. Rats treated with LINGO-1 siRNAs NPs showed overall better motor activity and coordination and a more compact myelin sheath histologically, illustrating the effectiveness of this approach [187]. Drug repurposing has also been explored, with anti-inflammatory cancer drug lenalidomide delivered in combination with in anti-oxidant cerium oxide NPs, and antibiotic minocycline was encapsulated in PEG liposomes, both of which result in improved clinical scores in EAE, although the cellular target in these studies was not further investigated [190,191].

Moreover, the specific targeting of macrophages for siRNA delivery has been investigated in a recent study where the pathologic crosstalk between pro-inflammatory macrophages and auto-reactive Th1/Th17 T cells was tackled by silencing the transcription factor c-Rel [161]. C-Rel plays a key role in inducing pro-inflammatory cytokine secretion by macrophages and therefore in controlling the T cell response, with a siRNA encapsulated into PEG-PLL-PLLeu MPs and tested in vitro on macrophages [161]. Decreased levels of secreted IL-1β among others were observed, suggesting that c-Rel silenced macrophages might be less able to induce Th1 and/or Th17 responses. Intraperitoneal (IP) injections of PEG-PLL-PLLeu-c-Rel MPs reduced the clinical score in the EAE model, and the fact that the MPs were preferentially localised in macrophages suggested that they played a crucial role in this phenotypic effect. Moreover, this was associated with a decrease of the number of infiltrating macrophages in the spinal cord and brain and lower serum levels of both IFNγ and IL-17A, suggesting that these MPs are able to dampen the Th1/Th17 response [161]. Together these studies illustrate the utility of nanotechnology for the delivery of novel therapeutics in MS models, with the latter showing the successful targeting of macrophages to induce a therapeutic response.

## 4. Optimising Delivery and NP/MP Uptake in Macrophages

As discussed, macrophages offer untapped potential in terms of therapeutic manipulation in MS, with nanotechnology as a promising means to realise this. While it is clear that macrophages are highly receptive to NP/MP mediated delivery, it is desirable to maximise the specificity of targeting to a therapeutically relevant population of cells, thus maximising the NP/MP payload and minimizing off target effects. In the functional modulation of macrophages in MS, specific targeting to M1-like macrophages in the CNS or their peripheral precursors could minimize the effects of this treatment on peripheral and other tissue macrophages, which are required in normal innate and adaptive immune defence. Here we explore the enhancement of macrophage targeting through particle size and functionalisation, which could prove useful in the development of strategies for macrophage targeting in MS and associated models.

### 4.1. Size

Particle size is a key biophysical characteristic affecting cellular targeting and uptake. While endocytosis and pinocytosis facilitate the entry of NP around 200 nm or smaller into most cell types, phagocytosis can accommodate the uptake of particles up to 10μm in diameter. This process is restricted to professional APCs, and thus larger particle sizes can be used to passively target macrophages, as reviewed extensively in [192]. In addition to restricting particle entry to phagocytosing cells, uptake studies show a trend for preferential uptake of larger NPs and MPs in macrophages compared with smaller NPs. In vivo studies with liposomes in both alveolar macrophages and in an atherosclerosis model show increased uptake with increasing size up to 2 μm and 500 nm respectively [193,194]. A similar trend is observed in vitro with PLGA and chitosan particles, with an optimal size at approximately 2 μm, and the latter study showing a second peak at 430 nm [195,196].

Despite the preference for larger NP/MP sizes, particles of smaller size are still frequently used for macrophage delivery [162,197,198,199,200], and may be more appropriate for a number of reasons. Administration route can factor in; nanomedicines delivered intravenously (IV) are restricted by small capillary size to avoid embolism [201], while small sizes are also preferred for intranasal administration for local CNS delivery (the nose to brain (NTB) route),which are restricted by axon diameter [202,203]. Additionally, for CNS entry from systemic circulation, smaller particles may have easier access across the BBB [201]. It is worth considering in designing strategies to target pro-inflammatory macrophages, that there is evidence that polarisation both contributes to size related uptake preferences of macrophages and can itself be influenced by NP/MP uptake. The investigation of silica NP (26 and 41 nm) and latex MP (1.75 μm) uptake by polarised cells indicates that while there is no significant difference in MP uptake, M1-like cells show significantly lower NP uptake than M2-like cells, proposed as a result of a higher endocytic capacity of M2-like cells [204]. Furthermore, there is evidence that smaller particles can increase the production of pro-inflammatory cytokines to a greater extent than larger particles [196].

### 4.2. Functionalisation

Enhancement of macrophage specific delivery can be achieved by the conjugation of ligands or antibodies to target highly expressed surface receptors on these cells. Mannose and galactose ligands have been exploited for their ability to bind the mannose receptor (CD206) and the galacto-type lectin, respectively. These receptors are highly expressed on macrophages and have been demonstrated for macrophage targeting in many disease contexts, among them infection [205,206], inflammatory bowel disease [207,208], cancerous tumours [209,210] tuberculosis [211] and atherosclerosis [212]. Notably, the use of mannose functionalised NPs has been demonstrated for the delivery of an antiretroviral drug targeted to macrophages in the CNS of rats, showing increased CNS drug concentrations following IV delivery compared with unmodified NPs or free drug [197].

The CD11b integrin is present on many leukocytes and is highly expressed in macrophages, their monocyte precursors, and microglia. A proof of concept study encapsulating leukaemia inhibitory factor (LIF) showed significantly increased therapeutic impact with anti-CD11b functionalisation in a myeloid cell line with M1-like characteristics, indicative of increased uptake [213]. In terms of microglial targeting, in a mixed glial culture model, anti-CD11b conjugation has been shown to maximise microglial uptake, while reducing the proportions of astrocytes and oligodendrocytes internalising NPs [214]. A separate study comparing anti-CD11b conjugation to that of non-specific IgG conjugation, showed that in vitro transfection efficiency of microglia with a microRNA cargo was increased from ~52% (IgG) to ~71% (CD11b) [200].

Additionally, CD64 may represent a promising candidate for mediating specific NP/MP delivery. CD64 or Fc γ receptor I (FcγRI) is constitutively expressed on monocytes and macrophages and inducible in neutrophils, but has shown to be substantially upregulated in macrophages with a pro-inflammatory or M1-like phenotype [215,216]. CD64 is also an MS relevant target, evidenced by its upregulation in macrophages of human disease samples [54]. As an NP/MP targeting ligand, in vitro macrophage uptake following conjugation of a CD64 antibody to solid lipid and PLGA NPs has been demonstrated, intended for future use in rheumatoid arthritis models [217,218]. Furthermore, Yong et al. demonstrate the utility of CD64 as a monocyte specific targeting ligand in a study delivering siRNA cargo to human peripheral blood mononuclear cells [219].

### 4.3. Macrophage Modulation: A Peripheral or CNS Centric Approach?

Worth considering in MS is the most pertinent location for targeted drug delivery to macrophages and how this relates to NP/MP design and administration. While macrophages that enter as monocytes in the hallmark immune infiltrate are capable of directly inflicting pathogenic damage as outlined above, peripheral monocytes also have a significant role in shaping the overarching immune landscape in which disease occurs, contributing to the cytokine environment and the shaping of T cell responses. An outstanding question thus remains as to whether targeting macrophages in the CNS or focusing delivery to peripheral monocyte precursors is the more appropriate therapeutic approach.

Peripheral monocyte uptake is readily achieved following systemic administration, which sees substantial NP uptake by circulating monocytes and DCs of the mononuclear phagocyte system (MPS) and potential accumulation in the spleen, liver, and kidneys owing to high numbers of those cells therein [220]. Manipulation of these cells may impact MS/EAE outcome by altering the immune response in the periphery, as evidenced in the aforementioned immune tolerance approaches [168,169,179] as well as in preventing CNS accumulation as a consequence of depletion [46,47,48]. Outside of immune tolerance approaches, however, peripheral modulation is not without concern owing to the potential for global immunosuppression. Directly targeting macrophages that have been mobilised to the CNS has the potential to avoid this complication, however, it represents a more complex target for drug delivery.

Systemically administered nanomedicines face two major hurdles before even considering their function in the brain; they typically must avoid uptake by peripheral macrophages in order to reach the CNS and also contend with the BBB, a highly selective barrier effected through the presence of tight junctions, degradative enzymes and selective transport proteins present where CNS microvessels interface with astrocytes [221]. With liposomes and hydrophobic carriers like PLGA, peripheral phagocyte evasion is most commonly achieved by polyethylene glycol (PEG) modification. PEG prolongs NP circulation by forming a hydrophilic layer around the NP and reducing opsonisation [220]. After overcoming peripheral uptake, most NP carriers, including PLGA, still cannot enter the CNS without the conjugation of ligands that exploit existing BBB transport mechanisms. Adsorptive-mediated transcytosis offers passage through the BBB by charge mediated binding of ligands to the negatively charged brain endothelial surface. PEG and also TAT, the HIV cell-penetrating peptide, have been used as ligands NP/MP targeted to macrophages within the CNS [222,223]. Receptor-mediated transcytosis offers a highly specific transport mechanism through the binding of conjugated ligands to specific receptors. With respect to CNS macrophage targeting, lactoferrin [222], transferrin receptor binding peptide [224], rabies virus glycoprotein [225], and mannose [197] are among those that have been explored.

Alternatively, localised CNS delivery can be achieved through the more recently investigated NTB route (fully reviewed in [226,227,228]), which notably bypasses the BBB. This may avoid the seeming contradiction in systemic delivery associated with trying to avoid phagocytosis in the periphery, while trying to target this property in CNS. NTB is not without challenges, however, and small administration volumes, restricted particle size and NP mucoadhesive properties must all be considered. Promisingly, respective targeting of microglia and macrophages by NTB administered NP/MPs has been demonstrated in both an LPS induced neuroinflammation model of Parkinson’s Disease and HIV infection [198,229]. Regarding MS and associated animal models, the NTB route is under investigation in terms of both naked [230,231] and NP encapsulated therapeutics for both existing and novel therapautics, as explored above [185,187,232], and could offer a novel avenue for MS centric therapeutics.

## 5. Concluding Remarks

In conclusion, peripherally derived macrophages play a dominant role in MS onset, progression and repair. The aforementioned studies suggest that modulating the polarisation status of macrophages with NP/MP-loaded drugs to enhance an M2-“switched” state could represent a valid and partially unexplored area of research. It is worth noting that future studies embarking in NP/MP administration in MS should seriously consider assessing the macrophage uptake and polarisation states as it is highly likely that this phenomenon contributes to overall efficacy in disease progression.

## Figures and Tables

**Table 1:** Approved Disease Modifying Treatments for Multiple Sclerosis (MS) and evidence for their effects on monocytes and/or macrophages.

	Type	FDA Approval	Format (Oral/Injectable)	Mechanism of Action	Studies in Monocytes/Macrophages	Adverse Effects
**Interferon β**	Cytokine	1993	Injection (SC or IM)	- Type 1 Interferon - Effect in B and T cells - Reduction in BBB disruption [111,112]	- IL-27 production by myeloid cells suppresses Th17 differentiation in EAE [113,114] - Increased response to IL10 in human monocytes [115]	Flu-like symptoms [116]
**Glatiramer Acetate**	Synthetic Copolymer [E,K,A,Y]_n_	1995	Injection (SC)	- Shift from Th1 to Th2 responses - Increased foxp3+ Tregs - [117,118]	- Shift treated patient monocytes to type II antigen presenting cells—Th2 T cell responses [119,120,121] - Increased phagocytosis in rat microglia and human monocytes [122,123]	Injection site reaction [111]
**Natalizumab**	Anti-alpha-4 integrin	2003	IV infusion	- Prevent α4 integrin mediated T cell migration and CNS infiltration	- Reduced CNS accumulation of activated microglia and macrophages with early therapy in EAE [124] - Reduced pro-inflammatory mir-155 in patient monocytes [125]	PML risk, Allergic Reactions [108]
**Fingolimod**	Antagonist of sphingosine 1 phosphate receptor	2010	Oral	- Suppress lymphocyte migration from lymph nodes	- Microglial M2 polarisation in stroke model [126] - Alteration cytokine production in patient monocytes [127,128,129] - Reduced pro-inflammatory mir-155 in patient monocytes [125]	Cardiovascular complications [130]
**Teriflunomide**	dihydroorotate dehydrogenase inhibitor	2012	Oral	- Suppress rapid expansion of lymphocytes by inhibition of the pyrimidine *de novo* synthesis pathway	-	abnormal liver enzymes, gastrointestinal symptoms [131]
**Dimethyl Fumarate**	Fumaric Acid Ester	2013	Oral	- reduction of Th1 responses - Nrf2 activator - NfkB inhibitor	- Decreased monocyte infiltration in EAE [132,133] - Glycolysis inhibition in murine macrophages [134] - Decreased pro-inflammatory cytokines in EAE [135,136] - Decreased pro-inflammatory cytokines and mir-155 in patient monocytes [125]	gastrointestinal symptoms, abnormal liver enzymes, flushing [137]
**Alemtuzumab**	Anti-CD52	2014	IV infusion	- Depletion of mainly mature T and B lymphocytes, to a lesser extent monocytes and dendritic cells	-	Development of other autoimmune disease, Intracerebral haemorrhage (rare) [109,138]
**Mitoxantrone**	Chemotherapeutic agent	2003	IV infusion	- DNA topoisomerase inhibitor - Suppressed cell proliferation - Impaired antigen presentation [139]	- Reduced ex vivo migration capacity of patient monocytes [140]	Leucopoenia [141]
**Ocrelizumab**	Anti CD-20	2017	IV infusion	- Depletion of B cells - Note: the only FDA approved DMT for PPMS	-	Infusion related reaction, infections [142]

**Table 2:** NP/MP strategies in MS models with impact in macrophages.

Reference	NP/MP Chemistry	Size	Cargo	Functionalised	Route of Delivery	Model	Target Cells	Additional Points
[161]	PEG-PLL-PLLeu copolymers	not reported	c-Rel siRNA	-	IP	EAE	Macrophage	
[162]	inorganic-organic hybrid NP	60–80 nm	glucocorticoids	-	IP and IV(more effective)	EAE	Macrophage	
[163]	PEGylated liposome	<100 nm	Prednisolone	PEG	IV	EAE	*not specified*	liposomes were found mostly in macrophages, microglia and astrocytes
[164]	liposome	<100 nm	methylprednisolone	-	IV	EAE	*not specified*	Compared with free drug, only liposomal formulation resulted in significantly decreased CD68+ cells
[165]	liposome	*not reported*	methylprednisolone	short peptide fragments of ApoE or of β-amyloid	IV	EAE	*not specified*	
[166]	PEGylated liposome	95–120 nm	methylprednisolone	PEG + Glutathione	IV	EAE	*non specified*	Bigger reduction in disease score with the targeted vs non targeted liposome
[167]	PLGA	540 nm	(tNP) PLP (coated)	-	IV	EAE	APCs	Taken up by macrophages and DCs, most antigen presentation by DCs
[168]	PLGA	*not reported*	(tNP) PLP + rapamycin	-	SC prophylactic, IV peak disease	EAE	APCs	in vivo trafficking—IV -accumulation in liver and spleen most localisation to Macrophages and DCs in the spleen, but SC goes to the draining lymphnodes
[169]	PLGA	350–835 nm	(tNP) PLP	-	IV	EAE	APCs (Macrophage)	Immunofluorescence staining showing co localisation with F4/80 positive macrophages, lungs, spleen, lymph nodes
[170]	PLGA	80nm, 400 nm	(tNP) PLP	-	IV	EAE	APC’s (DCs)	Larger particles show better uptake in BMDCs
[171]	PLGA	400–1500 nm	(tNP) MOG (coated)	-	IV or SC	EAE	APCs	SC admin not effective, non-significant trend to bring on disease more quickly
[172]	Au	60 nm	(tNP) MOG + small molecule (ITE)	PEG(to stabilize)	IV or IP	EAE	DC	ITE ligand activates the aryl hydrocarbon receptor (Ahr), which can induce tolerogenic DCs. Observed Ahr activation in Macrophages in vivo
[173]	poly(ε-caprolactone)	300–600 nm range	(tNP) Recombinant human MBP	-	SC	EAE	APCs	Histological observation of no macrophage or T cell infiltration in treated animals
[174]	PLGA	200 nm	(tNP) MOG and IL-10	-	SC	EAE	APCs	Authors suggest that observed T cell anergy and inhibited lymphocyte proliferation is due to induction of tolerance in macrophages
[175]	Acetalated Dextran	*not reported*	(tNP) MOG and Dexamethasone	-	SC		APC’s (Macrophage)	Reduced macrophage GM-CSF and IL-17
[176]	PLGA	*not reported*	(tNP) MOG, Vitamin D3, TGFb, GM-CSF	-	SC	EAE	APCs	Macrophages have second highest MP uptake in axillary lymph after DC’s, while these cells show equal uptake in inguinal lymph nodes. Treatment results in decreases numbers of activated macrophages in CNS
[177]	PLGA	400–500 nm	(tNP) PLP	-	IV	EAE		Localisation to spleen, liver, and lung at 3, 6, and 18 h post injection, cleared by 24 h
[178]	polystyrene, PLGA	500 nm	(tNP) PLP	-	IV	EAE	Macrophage	SC did not work as well as IV admin, NP show localisation to spleen marginal zone macrophages and uptake via MARCO receptor
